# Supplementary material for: Electrochemical Sensing of Favipiravir with an Innovative Water-Dispersible Molecularly Imprinted Polymer Based on the Bimetallic Metal-Organic Framework: Comparison of Morphological Effects
Source: Biosensors (Basel). 2022 Sep 19;12(9):769. doi: 10.3390/bios12090769 (PMC9496828; doi:10.3390/bios12090769)
Supplement: Supplementary file 1 [file biosensors-12-00769-s001.zip › biosensors-1918918-supplementary-update.pdf]

biosensors

MDPI

Supporting Information

Electrochemical Sensing of Favipiravir with An Innovative Water-Dispersible Molecularly Imprinted Polymer Based on The Bimetallic Metal-Organic Framework: Comparison of Morphological Effects

Nevin Erk [1,*], Mohammad Mehmandoust [1] and Mustafa Soylak [2,3,4]

[1] Department of Analytical Chemistry, Faculty of Pharmacy, Ankara University, Ankara 06560, Turkey
[2] Department of Chemistry, Faculty of Sciences, Erciyes University, Kayseri 38039, Turkey
[3] Technology Research & Application Center (TAUM), Erciyes University, Kayseri 38039, Turkey
[4] Turkish Academy of Sciences (TUBA), Ankara 06670, Turkey
[*] Corresponding author: erk@pharmacy.ankara.edu.tr

1. Material

Neutec Pharm kindly supplied Favipiravir. Ind. Cobalt(II) nitrate hexahydrate ($Co(NO_3)_2{\cdot}6H_2O$), Nickel(II) nitrate hexahydrate ($Ni(NO_3)_2{\cdot}6H_2O$), 2-methylimidazole (CAS No: 693-98-1), and 1,2,4,5-Benzenetetracarboxylic acid (H_4btec), and chitosan were purchased from Merck Company (Darmstadt, Germany). L-ascorbic acid (99.0%), L-arginine (98.0%), L-cysteine (97.0%), glucose (99.5%), uric acid (99.0%), and dopamine (99.0%) were obtained from Sigma Aldrich company. All other chemicals were of analytical or HPLC grade and utilized as received. Britton-Robinson buffer (B-R) was used in all steps. Sera-Flex human blood plasma samples were also purchased from Dyna-Tek Industries Inc. (Lenexa, KS, USA).

2. Apparatus

The morphology and structure of MIP-Co/Ni@MOF were attained by field emission scanning electron microscopy (FESEM, Quanta FEG 450). The AFM analysis of the MIP-Co/Ni@MOF was performed using a VEECO Multimode 8 Atomic Force Microscope and Micromeritics Gemini VII surface area. A Fourier Transform Infrared spectrophotometer (Perkin Elmer, 400 FT-IR/FT-FIR spectrometer spotlight 400 imaging system, ATR) was used to gather the synthesized materials' formation information. The XRD pattern of MIP-Co/Ni@MOF was collected by a Bruker AXS D8 Advance X-ray Powder Diffractometer (Cuk_{α}: 1.54 Aº). Electrochemical characterization of MIP-Co/Ni@MOF/SPE was performed by cyclic voltammetry (CV), differential pulse voltammetry (DPV), electrochemical impedance spectroscopy (EIS), and chronoamperometry (CA) on SPE (DRP-110, where the working and auxiliary electrodes are made of carbon while the reference electrode is available in silver/silver chloride) using the PGSTAT128N model potentiostat/galvanostat in a one-compartment electroanalytical cell with NOVA 2.10 software.

3. Preparation of Co/Ni@MOF Nanosheets

Firstly, 3.0 mmol $Co(NO_3)_2{\cdot}6H_2O$ and 1.0 mmol $Ni(NO_3)_2{\cdot}6H_2O$ were dissolved in 30.0 mL of methanol (solution 1). Afterward, 3.0 mmol of 2-methylimidazole was dissolved in methanol (solution 2). Then, solutions 1 and 2 were mixed under stirring for 5.0 min. The mixture solution was left to stand for 24 h at an ambient temperature. Then, the as-synthesized yellowish precipitate was centrifuged, rinsed with pure methanol and water several times, and then dispersed in deionized water [1].

4. Preparation of Co/Ni@MOF Nanododecahedron

Briefly, 2.0 mmol, composed of $CoCl_2·6H_2O$ and $NiCl_2·6H_2O$ equivalently in 10.0 mL ultrapure water with 0.1 mM H_4btec and 2.5 mM NaOH, was poured into a 25.0 mL Teflon-lined stainless-steel autoclave after mixing and then heated up to 110 °C for 72 h. It was cooled down to ambient temperature, and after filtration the sample was rinsed several times with ultrapure water and dried for 24 h in an oven at 50 °C. Eventually, brown powders of Co/Ni-MOF nanododecahedron were collected [2].

5. Preparation of MIP-Co/Ni@MOF/SPE

In order to prepare MIP-Co/Ni@MOF, 0.6 g of chitosan was dissolved in 50 mL of acetic acid solution (1.0%, v/v) under magnetic stirring. Afterward, 0.6 mmol FAV were added, allowing the formation of precomplexes between chitosan and the template at room temperature for 4 h. In the next step, 0.3 g of Co/Ni@MOF (nanododecahedrons and nanosheets were added separately) was transferred to the above solution, then stirred vigorously for 18 h at ambient temperature. Afterward, 50 mL of 1.0 mol L^{-1} NaOH solution was added rapidly into the resultant solution and stirred for 4 h. Then, the obtained product was centrifuged to collect the black precipitate, followed by several washing with deionized water until a pH of 6.0 was reached, and was redispersed in 0.01 mol L^{-1} HCl for 10 min under ultrasonic irradiation. The pH of the solution was kept at less than 6.0 during the whole procedure to ensure the deprotonation of chitosan. Finally, MIP-Co/Ni@MOF was obtained after repeatedly eluting FAV with a methanol-acetic acid solution (9.0:1.0, v/v) two times until template molecules were removed and dried at 60 °C overnight [3]. The nonimprinted polymers (NIPs) were synthesized with the same procedure except in the absence of templates. The MIP-Co/Ni@MOF nanocomposite modified electrode was prepared by dropping 8.0 μL of the above suspensions onto the surface of the SPE and drying at room temperature. The electrode surface was rinsed with water to remove the unattached nanocomposite.

6. Assay Procedure for The Preparation of Real Samples

In order to produce the human plasma sample, a certain amount of human plasma samples was spiked with varying concentrations of FAV. These were separately placed in centrifuge tubes and filled to volume with B-R buffer (pH 4.0). Centrifugation at 8000 rpm for 10.0 minutes separated the precipitated proteins. Moreover, the urine samples were carefully filtered using PTFE syringe filter (0.45 m) membrane filters for urine sample processing. A particular amount of FAV solution was added to the urine solution to prepare FAV-spiked urine samples. Finally, the standard addition technique determined FAV in the above samples.

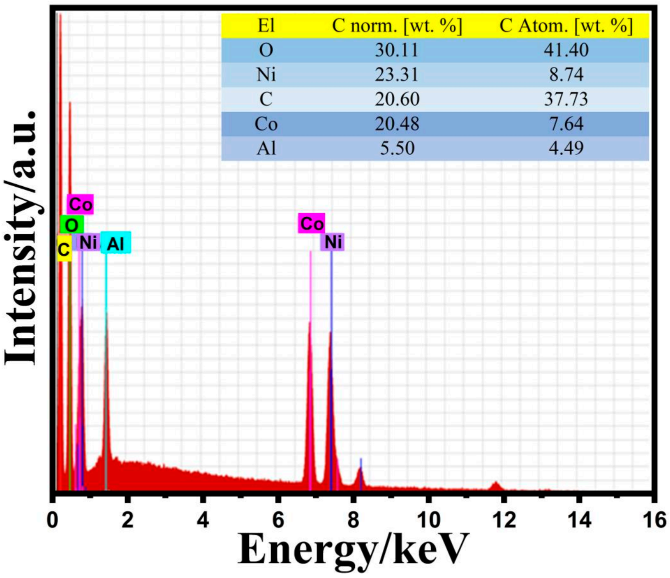

Figure S1. EDX spectrum of MIP-Co/Ni@MOF.

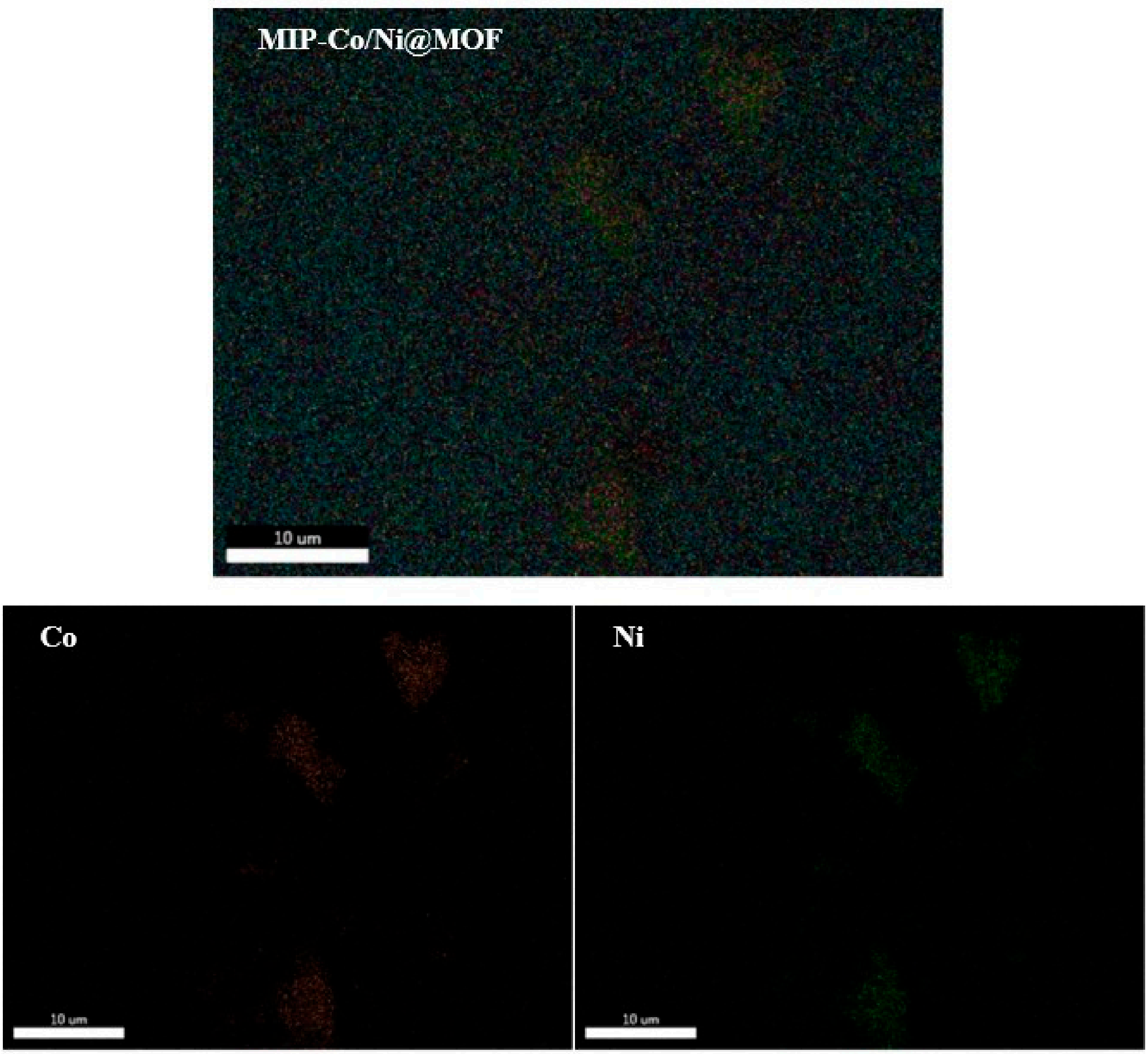

Figure S2. EDX mapping of MIP-Co/Ni@MOF.

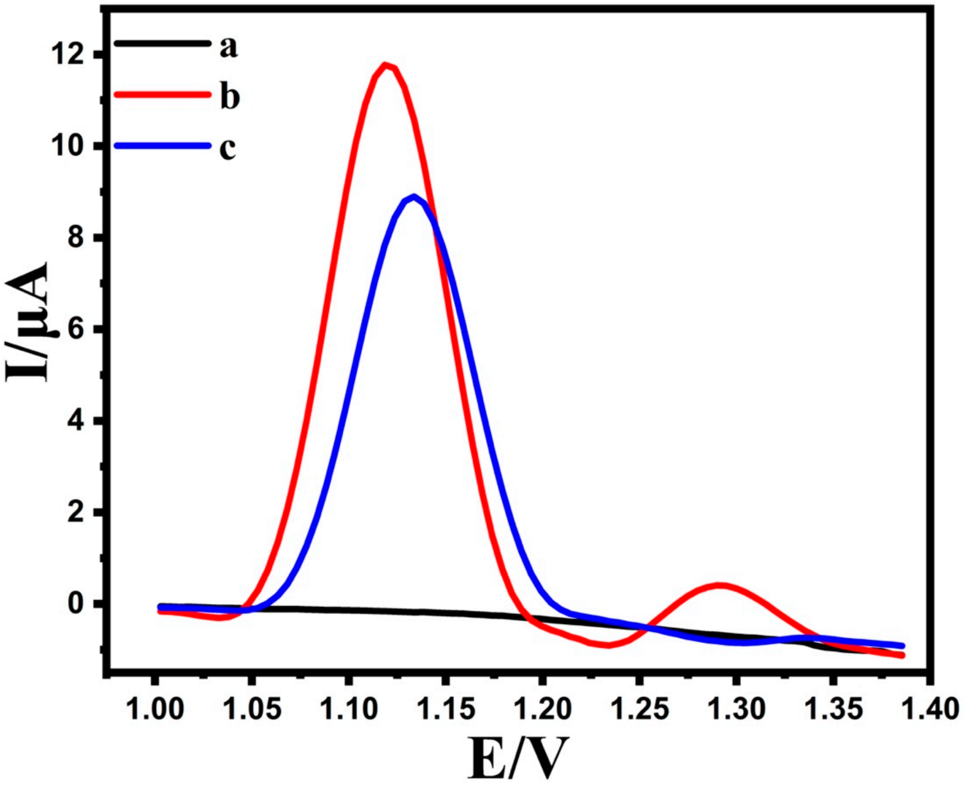

Figure S3. DPV of blank (a) and 0.05 μM FAV at MIP-Co/Ni@MOF nanosheets (b) and MIP-Co/Ni@MOF nanodedecahedron (c).

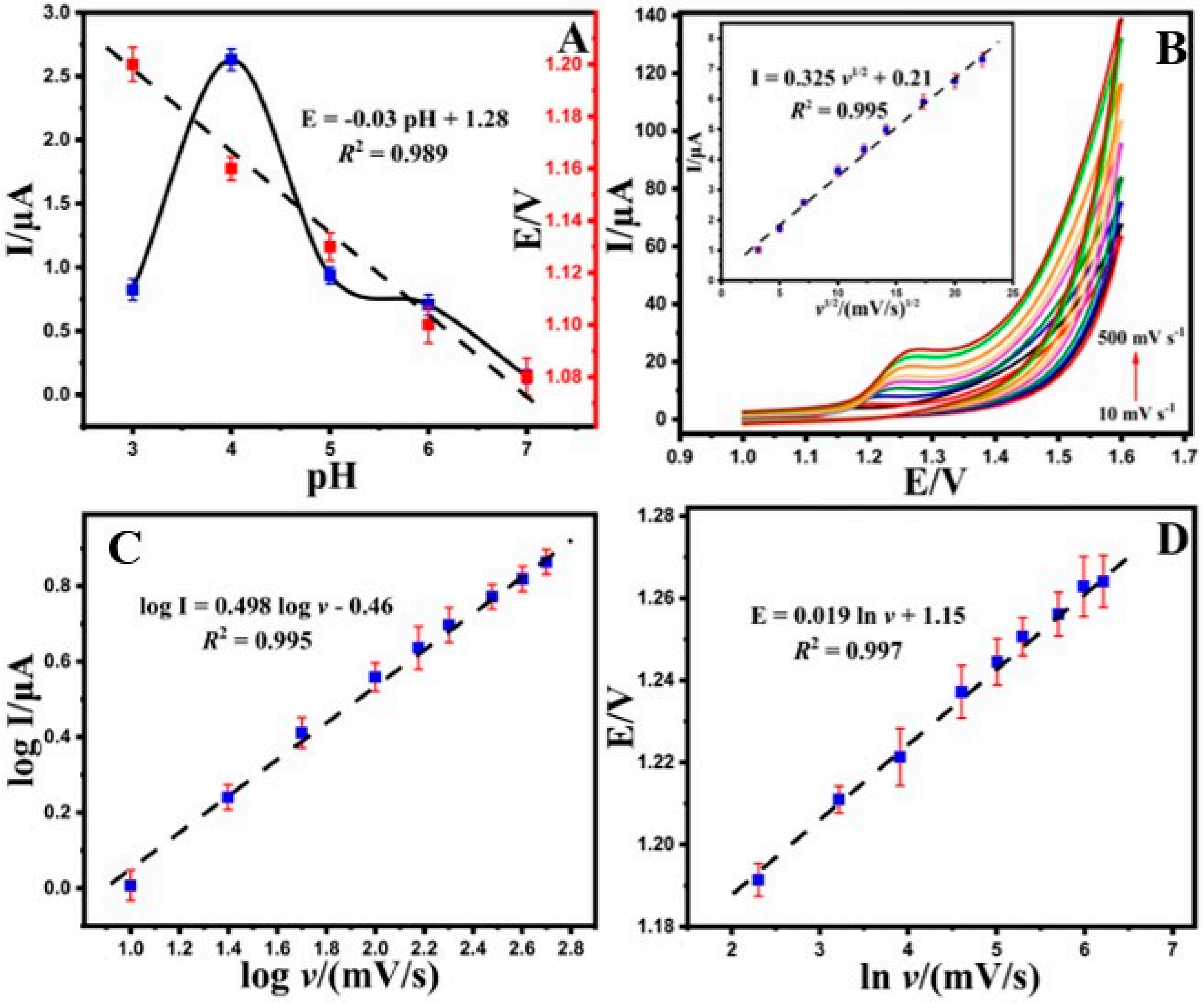

Figure S4. (**A**) The plot of the peak potentials versus pH and (red dot) and the plot of the peak currents versus pH (blue dot), (**B**) CVs of 0.05 μM FAV on MIP-Co/Ni@MOF/SPE in B-R (pH 4.0) at various scan rates of 10.0 to 500.0 mV·s^{-1} (inset). The plot of the square root of the scan rate and the peak current (**C**); The plot of the log I_{pa} vs. log v obtained at the surface of MIP-Co/Ni@MOF/SPE. (**D**) The relationship between E_{pa} vs. ln v at the surface of MIP-Co/Ni@MOF/SPE.

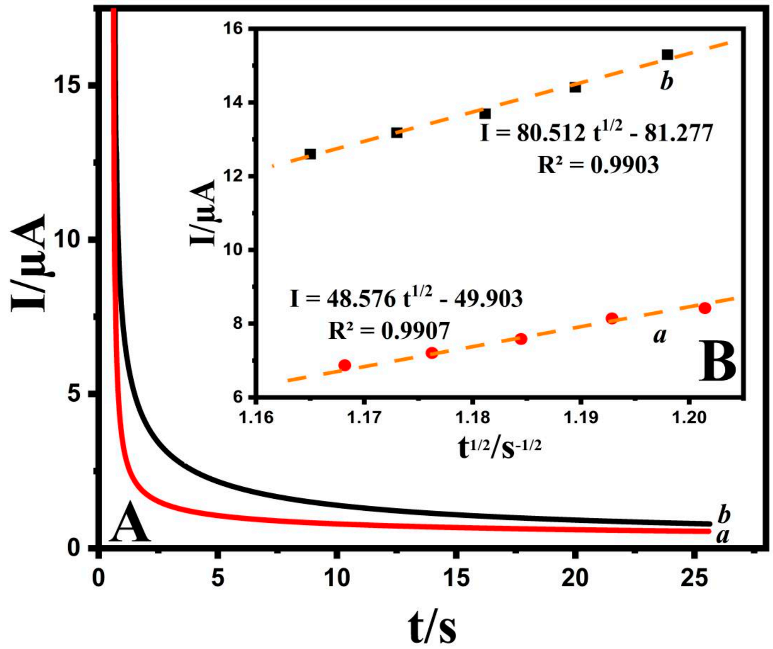

Figure S5. (**A**) Chronoamperograms obtained at MIP-Co/Ni@MOF/SPE in the presence of (a) 200 and (b) 300 μM FAV in B-R buffer (pH 4.0). (**B**) Cottrell`s plot for the data from the chronoamperograms.

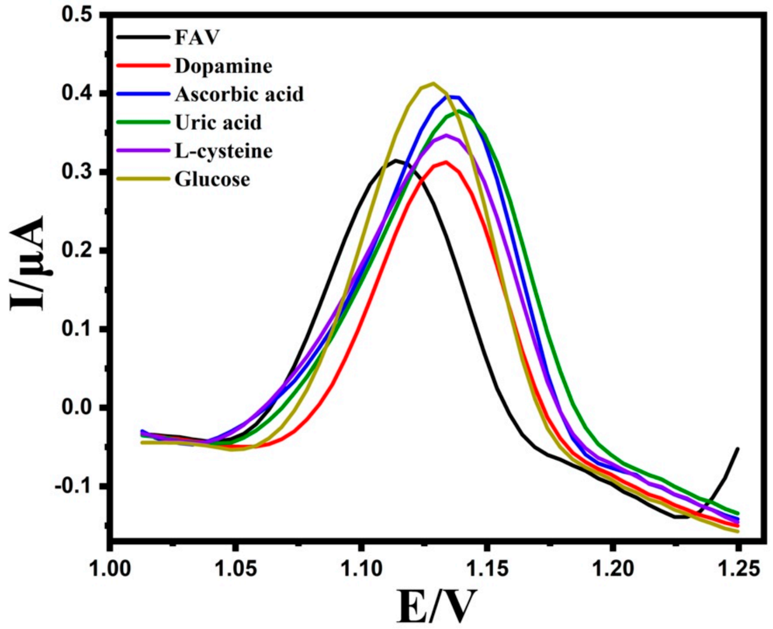

Figure S6. DPVs of FAV in the presence of interferences at MIP-Co/Ni@MOF/SPE.

References

1. Wang, M.; Wang, P.; Li, C.; Li, H.; Jin, Y. Boosting electrocatalytic oxygen evolution performance of ultrathin Co/Ni-MOF nanosheets via plasmon-induced hot carriers. *ACS Appl. Mater. Interfaces* **2018**, *10*, 37095–37102.
2. Ye, G.; Luo, P.; Zhao, Y.; Qiu, G.; Hu, Y.; Preis, S.; Wei, C. Three-dimensional Co/Ni bimetallic organic frameworks for high-efficient catalytic ozonation of atrazine: Mechanism, effect parameters, and degradation pathways analysis. *Chemosphere* **2020**, *253*, 126767.
3. Ostovan, A.; Ghaedi, M.; Arabi, M.; Yang, Q.; Li, J.; Chen, L. Hydrophilic multitemplate molecularly imprinted biopolymers based on a green synthesis strategy for determination of B-family vitamins. *ACS Appl. Mater. Interfaces* **2018**, *10*, 4140–4150.
